# The effects of vitamin B_12_ and diclofenac and their combination on cold and mechanical allodynia in a neuropathic pain model in rats 

**Published:** 2013

**Authors:** Esmaeal Tamaddonfard, Farzad Samadi, Karim Egdami

**Affiliations:** 1 *Department of Basic Sciences, Faculty of Veterinary Medicine, Urmia University, Urmia, Iran; *; 2 *Graduated, Faculty of Veterinary Medicine, Urmia University, Urmia, Iran.*

**Keywords:** Allodynia, Diclofenac, Rats, Tibial nerve injury, Vitamin B_12_

## Abstract

The present study was performed to investigate the effects of long-term intraperitoneal (IP) injection of vitamin B_12_ and diclofenac in separate and combined treatments on cold and mechanical allodynia in a neuropathic pain model in rats. Neuropathic pain was induced by crush injury in right tibial nerve. Acetone spray and von Frey tests were used to obtain cold and mechanical allodynia responses, respectively, on day 11 after nerve crush. Normal saline, vitamin B_12_ and diclofenac were injected intraperitoneally for 10 consecutive days after surgery. Normal saline treated rats showed cold and mechanical allodynia responses after nerve crush. Vitamin B_12_ at doses of 50, 100 and 200 µg kg^-1^ and diclofenac at a dose of 2 mg kg^-1^ produced antiallodynic effects. Antiallodynic effects were not observed when subanalgesic doses of vitamin B_12_ (25 µg kg^-1^) and diclofenac (0.25 mg kg^-1^) were used together. By increasing the dose of vitamin B_12_ to an effective dose (100 µg kg^-1^), antiallodynic effects were observed when compared with diclofenac (0.25 mg kg^-1^) alone. The results indicated that vitamin B_12_ and diclofenac produced neuropathic pain suppressing effects. Moreover, a potentiation effect was observed between vitamin B_12_ and diclofenac.

## Introduction

Neuropathic pain is a consequence of nerve injury characterized by the presence of exaggerated responses to painful stimuli (hyperalgesia), pain response to normally innocuous stimuli (allodynia) and spontaneous pain.^1^ These abnormal pain sensations have been associated with various complex physiological changes in the peripheral and central nervous system.^2^ The pharmaco-therapy for neuropathic pain has had a limited success with little or no response to commonly used pain reducing drugs such as non-steroidal anti-inflammatory drugs (NSAIDs) and opiates.^3^ Consequently, there is a considerable need to explore novel treatment modalities. 

In the search for alternatives, B vitamins have been found to be a useful pharmacological tool for treating peripheral neuropathy and related pain signs.^4^^,^^5^ The pain reducing effects of acute treatments with vitamin B_12 _alone or in combination with other B vitamins including vitamins B_1_ and B_6_ have been reported in sciatic nerve crush, spinal nerve ligature and diabetic models of neuropathic pain in rats and mice.^6^^-^^8^

Diclofenac, naproxen, rofecoxib and celecoxib, as NSAIDs are among the most widely used medications in the world because of their demonstrated efficacy in reducing pain and inflammation.^9^ However, treatment with NSAIDs may be accompanied by adverse effects such as gastrointestinal damage, platelet dysfunction, convulsions, disorientation, hallucination, and loss of consciousness.^10^

Some interactions exist between B vitamins and NSAIDs in modulation of pain. Garcia-Reyes *et al*. showed that the combination of diclofenac and B vitamins produced a better antihyperalgesic effect in carrageenan-induced hyperalgesia,^11^ whereas Granados-Soto *et al*.^12^ reported that diclofenac did not further increase vitamin B_12_-induced antiallodynia. The present study was aimed to investigate the effects of long-term separate and combined IP administrations of diclofenac and vitamin B_12_ on allodynia in tibial nerve-crushed rats. Tibial nerve is an important branch of sciatic nerve, and experimentally-induced injury in tibial nerve has been established as a neuropathic pain model in rats.^13^^-^^15^

## Materials and Methods

**Animals. **Healthy adult male Wistar rats, weighing 220–240 gram were used in this study. Rats were maintained in groups of 6 per cage in a light-dark cycle (light on at 07:00 h) at a controlled ambient temperature (22 ± 0.5 ˚C) with *ad libitum* food and water. Six rats were used for each experiment. All experiments were performed between 13:00 and 17:00. All research and animal care procedures were approved by the Veterinary Ethics Committee of the Faculty of Veterinary Medicine of Urmia University and were performed in accordance with the National Institutes of Health Guide for Care and Use of Laboratory Animals.^16^^,^^17^

**Drugs. **Drugs used in the present study included vitamin B_12_ and diclofenac sodium. Drugs were purchased from Sigma-Aldrich Chemical Co. (St. Louis, MO, USA). All drugs were dissolved in sterile normal saline 30 min before IP injections.

**Grouping.** The animals were randomly divided into following groups of six rats each: 

Group 1: This group was received IP injection of normal saline for 10 consecutive days and served as intact (control) group. 

Group 2: This group was received IP injection of normal saline for 10 consecutive days after surgery without induction of crush injury in tibial nerve and served as sham surgery group. 

Group 3: In this group IP injection of normal saline was done for 10 consecutive days after surgically-induced crush injury in tibial nerve and served as crush plus normal saline group.

Groups 4, 5, 6, 7: These groups were received IP injection of vitamin B_12_ at doses of 25, 50, 100 and 200 µg kg^-1^, respectively, for 10 consecutive days after surgically-induced crush injury in tibial nerve and served as crush plus vitamin B_12_ groups.

Groups 8, 9, 10, 11: In these groups IP injection of diclofenac was performed at doses of 0.25, 0.5, 1 and 2 mg kg^-1^, respectively, for 10 consecutive days after surgically-induced crush injury in tibial nerve and served as crush plus diclofenac groups.

Groups 12: This group was received IP co-administration of subanalgesic doses of vitamin B_12_ (25 µg kg^-1^) plus diclofenac (0.25 mg kg^-1^) for 10 consecutive days after surgically-induced crush injury in tibial nerve. 

Group 13: In this group IP co-administration of an analgesic dose of vitamin B_12 _(100 µg kg^-1^) and diclofenac (0.25 mg kg^-1^) was performed for 10 consecutive days after surgically-induced crush injury in tibial nerve. 

The protocol for this study, including doses of vitamin B_12_ and diclofenac were designed according to previous studies in which 0.02-2 mg kg^-1^ once and 0.5 mg kg^-1^ for 7-14 days of vitamin B_12_ and 1-10 mg kg^-1^ once and 5 mg kg^-1^ for 7-14 days of diclofenac were used.^7^^,^^18^^,^^19^

**Surgical procedure. **Rats were anesthetized by IP injection of a mixture of ketamine (80 mg kg^-1^) and xylazine (10 mg kg^-1^). The area above the right lower thigh was prepared aseptically. A 2-cm incision was made over the lateral aspect of the hind limb, and muscles were bluntly dissected in order to expose the tibial nerve. The nerve was crushed at 2-3 mm distal to sciatic nerve trifurcation point using a small hemostatic forceps for a period of 60 sec.^20^ The crushed zone was approximately 3-4 mm^2 ^and uniformly transparent for several minutes thereafter. In sham-operation group, the tibial nerve was exposed but not crushed. The muscle layers were closed using 4/0 chromic gut sutures, and skin was closed with 3/0 silk sutures.

**Cold allodynia. **Cold allodynia was measured as the number of foot withdrawal responses after application of cold stimuli to the plantar surface of hind paw.^21^ One drop of 100% acetone was gently applied to the mid-plantar surface of the rat with a syringe connected to a thin polyethylene tube. A brisk foot withdrawal response after the spread of acetone over the plantar surface of the paw was considered as a sign of cold allodynia. The testing was repeated 10 times with an interval of approximately 3-5 min between each test. The response frequency to acetone was expressed as a paw withdrawal frequency (PWF) ([number of paw withdrawals/number of trails] × 100). 

**Mechanical allodynia. **Mechanical allodynia was assessed using an electronic von Frey Anesthesimeter (IITC-Life Science Instruments, Woodland Hill, CA) as described by Caplan *et al*.^22^ Briefly, the rats were placed in individual plexiglass chambers (18 × 10 × 20 cm) with wire mesh floor, and allowed to explore and groom until they settled down. A set of von Frey filaments with the TBA value is generally regarded as a remarkable indicator for determining of deterioration of the organoleptic bending force ranging from 1-60 *g* (No. 5-15, respectively) were applied in an ascending order to the plantar surface of the right hind paw. Hind paw withdrawal was considered as positive response. The stimulation with one filament was repeated five times at 10-15 sec intervals and where there was no response the next filament with greater bending force was applied. The lowest force required to elicit a paw withdrawal response was recorded as the paw withdrawal threshold (PWT) (*g*). 

**Statistical analysis. **All data were analyzed using one-way ANOVA followed by Duncan’s test. All the values are expressed as the mean ± SEM. Statistical significance was set at *p* < 0.05.

## Results

**Fig. 1:**
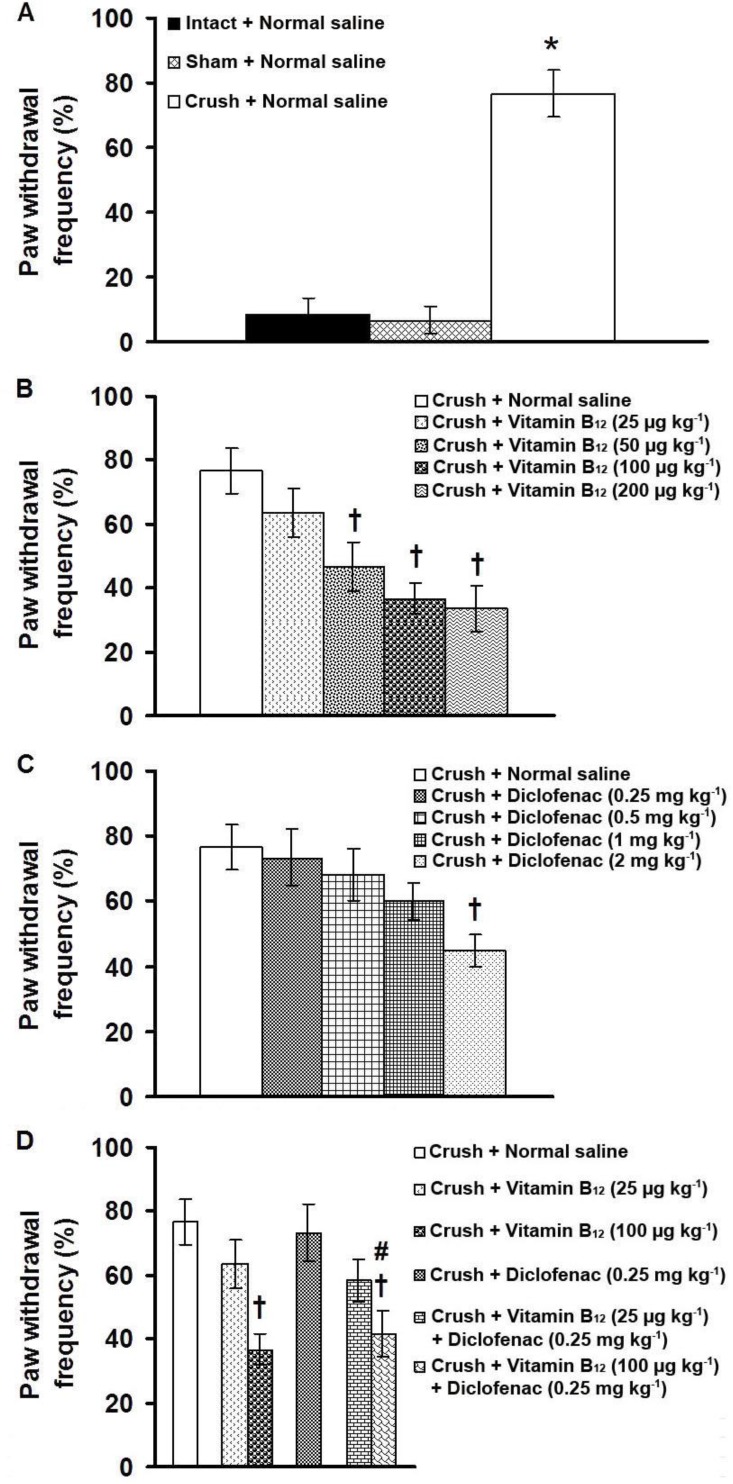
Paw withdrawal frequency (cold allodynia) induced by acetone test in tibial nerve-crushed rats (A) and the effects of long-term administration of vitamin B_12_ (B), diclofenac (C) and vitamin B_12_ plus diclofenac (D) on paw withdrawal frequency.

^⋆^ *p* < 0.05 as compared with intact and sham groups.

^†^ *p* < 0.05 as compared with crush plus normal saline treated group.

^#^ *p* < 0.05 as compared with diclofenac (0.25 mg kg^-1^) treated group.

Figure 1 shows the effects of separate and combined IP injections of vitamin B_12_ and diclofenac on percentage of paw withdrawal frequency induced by application of acetone in plantar surface of tibial nerve-crushed rats. Plantar surface application of acetone in intact and sham-operated animals produced negligible paw responses. Significant differences in paw withdrawal frequency were observed among intact, sham-operated and crush groups (Fig. 1A). Long-term IP injections of vitamin B_12_ at doses of 50, 100 and 200 µg kg^-1^, but not at a dose of 25 µg kg^-1^, significantly reduced paw withdrawal frequency (F_(4,25)_= 6.942, *p* < 0.05, one-way ANOVA, Fig. 1B). Diclofenac at doses of 0.25, 0.5 and 1 mg kg^-1^ produced no significant effect, but at a dose of 2 mg kg^-1^, diclofenac significantly lowered paw withdrawal frequency (F_(4,25)_= 2.927, *p* < 0.05, one-way ANOVA, Fig. 1C).

No significant antiallodynic effects were observed when a combination treatment was performed with subanalgesic doses of vitamin B_12_ (25 µg kg^-1^) and diclofenac (0.25 mg kg^-1^). Co-administration of an effective dose of vitamin B_12_ (100 µg kg^-1^) with a subanalgesic dose of diclofenac (0.25 mg kg^-1^) produced an antiallodynic effect as compared with 0.25 mg kg^-1 ^of diclofenac (F_(3,20)_ = 8.567, *p* < 0.05, one-way ANOVA, Fig. 1D).

**Fig. 2:**
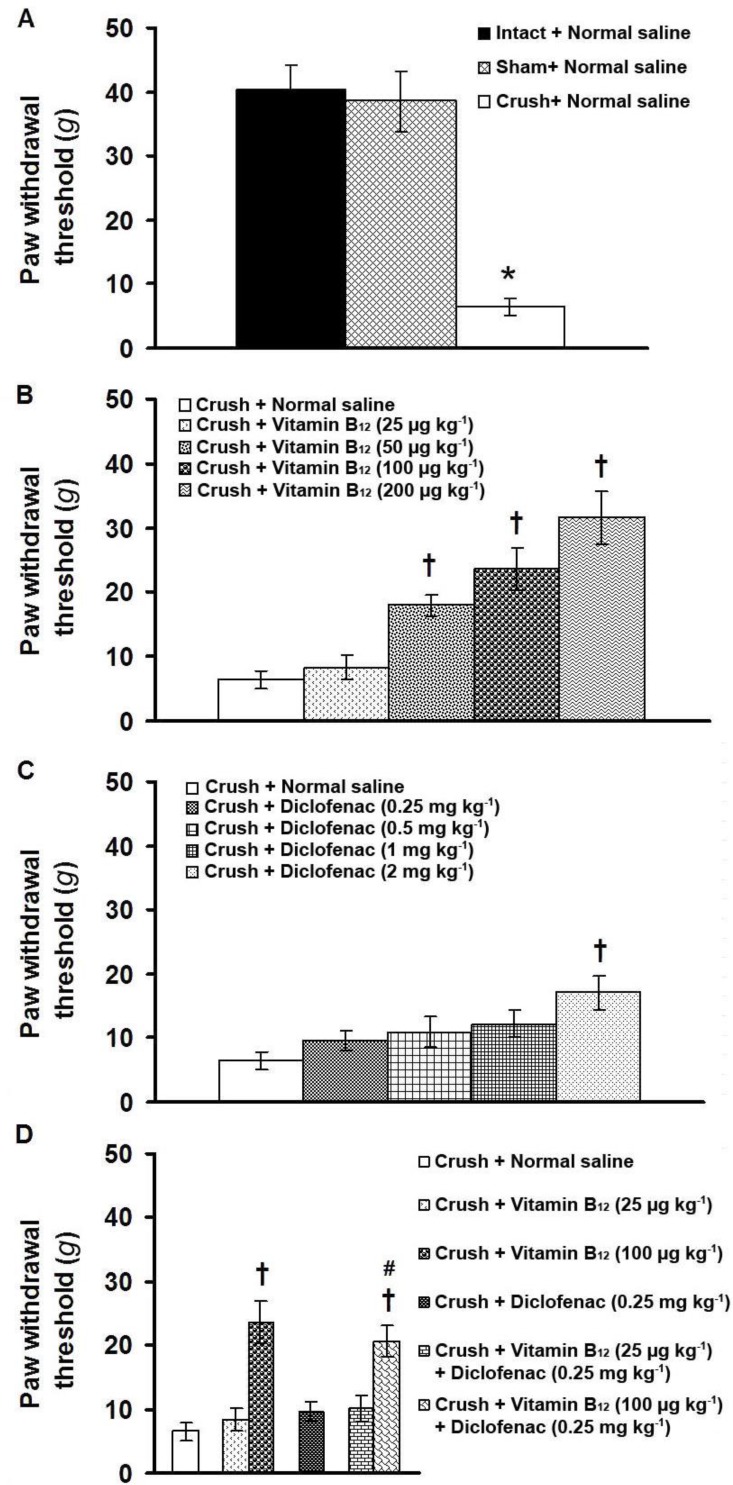
Paw withdrawal threshold (mechanical allodynia) induced by von Frey test in tibial nerve-crushed rats (A) and the effects of long-term administration of vitamin B_12_ (B), diclofenac (C) and vitamin B_12_ plus diclofenac (D) on paw withdrawal threshold.

^⋆^ *p* < 0.05 as compared with intact and sham groups.

^†^ *p* < 0.05 as compared with crush plus normal saline treated group.

^#^ *p* < 0.05 as compared with diclofenac (0.25 mg kg^-1^) treated group.

Figure 2 shows the effects of separate and combined IP injections of vitamin B_12_ and diclofenac on paw withdrawal threshold induced by application of von Frey filaments in plantar surface of tibial nerve-crushed rats. Plantar surface application of von Frey filaments in intact and sham-operated animals produced negligible paw responses. A significant (*p* < 0.05) allodynia was observed when crush group was compared with intact and sham-operated groups (Fig. 2A). Long-term IP injections of vitamin B_12_ at doses of 50, 100 and 200 µg kg^-1^, but not at a dose of 25 µg kg^-1^, significantly increased paw withdrawal threshold (F_(4,25)_= 15.435, *p* < 0.05, one-way ANOVA, Fig. 2B). Diclofenac at the doses of 0.25, 0.5 and 1 mg kg^-1^ produced no significant effect, but at a dose 2 mg kg^-1^, diclofenac significantly increased paw withdrawal threshold (F_(4,25)_= 3.424, *p* < 0.05, one-way ANOVA, Fig. 2C). Co-administration of subanalgesic doses of vitamin B_12_ (25 µg kg^-1^) and diclofenac (0.25 mg kg^-1^) did not produce any antiallodynic effects (Fig. 2D). Co-administration of an effective dose of vitamin B_12_ (100 µg kg^-1^) with a subanalgesic dose of diclofenac (0.25 mg kg^-1^) produced an antiallodynic effect compared with 0.25 mg kg^-1 ^of diclofenac (F_(3,20)_= 11.924, *p* < 0.05, one-way ANOVA, Fig. 2D). 

## Discussion

In the present study, cold and mechanical allodynia were produced after induction of tibial nerve crush injury. Tibial nerve injury model is a novel, surgically uncomplicated, rat model of neuropathic pain based on a unilateral transection (neuroctomy) of the tibial branch of the sciatic nerve.^14^ Some researchers have demonstrated that partial axotomies of one distal nerve (tibial nerve ligation) is more effective than total axotomies at producing hindpaw mechanical and thermal pain behaviors.^15^^,^^23^ Tibial nerve injury is induced by needlestick using 30G, 22G and 18G needles which produce cold and mechanical allodynia that is assessed by acetone spray and calibrated mono-filaments, respectively.^24^

The results reported here indicated that long-term administration of vitamin B_12_ alleviated cold and mechanical allodynia induced by tibial nerve crush injury. In dorsal root compression model of neuropathic pain, vitamin B_12_ at high doses and in combination with vitamins B1 and B6, without any effect on mechanical hyperalgesia, reduced thermal hyperalgesia.^18^ Moreover, long-term administration of vitamin B_12_ in combination with vitamins B_1_ and B_6_ suppressed the signs of pain in streptozotocin-induced diabetic rats.^7^ Granados-Soto *et al*.^12^ reported an antiallodynic effect of vitamin B_12_ in L_5_ and L_6_ spinal nerve ligation model of neuropathic pain in rats. The antinociceptive effects induced by B vitamins may be dependent on their action in the central nervous system. The increased activity of dorsal horn neurons induced by electrical stimulation of C fibers is reduced by B_1_, B_6_ and B_12 _The results of the present study showed that long-term administration of diclofenac, only at high dose (2 mg kg^-1^) attenuated tibial nerve crush induced cold and mechanical allodynia. It is known that diclofenac, as other non-selective NSAIDS, is able to impair prostaglandin synthesis by inhibition of the cyclooxygenase isozymes COX-1 and COX-2 in both, the injured tissues and the central nervous system.^28^ One time subcutaneous injection of diclofenac did not modify spinal nerve ligation-induced allodynia in rats.^12^ In the rat brachial plexus avulsion model of neuropathic pain, diclofenac failed to attenuate both mechanical and cold allodynia.^29^ However, in chronic constriction nerve injury model of neuropathic pain in rats, intrathecal injection of diclofenac with a minimal effect on mechanical allodynia, exacerbated thermal hyperalgesia.^30^ The differences between diclofenac effects may be related to the route of administeration of diclofenac and model of neuropathic pain used.

Administration of the high dose of NSAIDs such as diclofenac produce several adverse reactions, primarily gastrointestinal toxicity such as hemorrhages and ulceration.^31^ One might expect to eliminate this toxicity by a strategy, which provides an effective treatment with a dose of NSAIDs as low as possible. In the present study, sub-analgesic doses of vitamin B_12_ and diclofenac produced no effect on neuropathic pain signs when used together. By increasing the dose of vitamin B_12_ to an effective dose, the subanalgesic dose effect of diclofenac was converted to an effective action. These mean that vitamin B_12_ potentiated the effect of diclofenac in attenuating neuropathic pain symptoms. The experimental and clinical uses of combinations of analgesic agents have increased significantly in the last few years. The purpose is to associate two or more drugs with different mechanisms of action in hope of achieving a synergistic interaction that yields a sufficient analgesic effect with low doses of each agent, therefore, reducing the intensity and incidence of untoward effects.^32^ It has been reported that diclofenac is not able to increase the antiallodynic effect of vitamin B_12_ in spinal nerve ligation model of neuropathic pain.^12^ It seems that Vitamin B_12_ increases the antinociceptive effects of diclofenac in other models of pain such as carrageenan-induced hyperalgesia and lower-limb fracture and surgery-induced acute pain.^11^^,^^33^ Mibielli *et al*. reported that combination therapy with B vitamins plus diclofenac was superior to diclofenac monotherapy in reducing the pain associated with inflammatory conditions in humans.^34^ Although the analgesic mechanisms of vitamin B_12_ and diclofenac are different, experimental data suggest the involvement of nitric oxide-cGMP-K^+^ channels pathway and nitric oxide-cGMP system for antinociceptive effects of diclofenac and vitamin B_12_, respectively.^35^^,^^36^ However, other mechanism have been proposed, like capability of vitamin B_12_ in blocking the activation of COX-2 in experimental colitis.^37^ The real mechanisms involved in the potentiation for the combination await future elucidation.

In conclusion, the results of the present study showed that vitamin B_12_ and diclofenac (at a high dose) produced antiallodynic effects in tibial nerve crush injury model of neuropathic pain in rats. Vitamin B_12_ potentiated the effect of diclofenac on cold and mechanical allodynia. The inhibition of cyclooxygenase may be involved in the antiallodynic effect of vitamin B_12_.
